# Research on Properties of Dopamine and Silicon Carbon Black Modified Basalt Fiber Reinforced Magnetorheological Elastomer

**DOI:** 10.3390/polym14193949

**Published:** 2022-09-21

**Authors:** Shaoqiang Wang, Tianbao Liu, Yi Li, Ce Liang

**Affiliations:** 1 School of Computer Science and Technology, Changchun University, Changchun 130022, China; 2 Key Laboratory of Automobile Materials, Ministry of Education, College of Materials Science and Engineering, Jilin University, Changchun 130025, China

**Keywords:** magnetorheological elastomer precursors, silicon carbon black, basalt fiber, dopamine, surface modification

## Abstract

The basalt fibers (BF) and the basalt fibers etched by H_2_SO_4_ (BF^H^) were modified by polydopamine (PDA) or synergistically modified by PDA and silicon carbon black (SiCB). The effects of modified BF, BFH and SiCB on the basic mechanical properties and magnetorheological (MR) effects of natural rubber/butadiene rubber-based magnetorheological elastomer precursors (MREs) were investigated. The results show that the tensile strength, tear strength and stress at 300% strain of MREs/PDA-BF^H^-SiCB prepared with BF^H^ synergistically modified by PDA and SiCB reach the maximum values, which are 9.58 MPa, 24.07 kN/m and 4.13 MPa, respectively. Additionally, its MR effect is more than three times higher than that of MREs before composite modification.

## 1. Introduction

With the rapid development of science and technology, people have started to pay more attention to the application and development of smart materials. Magnetorheological elastomer precursors (MREs) are classified as smart materials because their properties can be changed with the applied magnetic field [1,2,3]. MREs consist of a non-magnetic matrix (usually elastomer) containing a suspension of permeable magnetic particles. Most MREs are made of rubber. With the development of the economy, people’s awareness of environmental protection has been growing stronger and stronger. As an indispensable part of life, rubber has also received more and more attention for its green and low-polluting production. Carbon black is the most common traditional filler for rubber. When carbon black is filled with rubber, it will greatly increase the performance of rubber, but it is a fossil fuel product, and the entire production process involves a lot of pollution and energy waste, which is inconsistent with green environmental protection. This problem encourages researchers to look for and develop potential natural green fillers to replace traditional carbon black for rubber reinforcement [4,5], so as to meet the requirements of green production. Silicon carbon black (SiCB), a kind of C-SiO_2_ biphasic filler prepared from waste rice husks produced by controlling pyrolysis conditions, is green and renewable [6]. Using it to replace the traditional filler is a feasible method. However, SiCB itself has poor dispersibility and is easy to agglomerate, and it is difficult for it to to be effectively wetted by rubber. Therefore, it is necessary to modify SiCB to enhance its dispersibility and combination with the matrix to improve its usability in the industry. In 1923, French national Paul Dhe [7] extruded fibers from basalt and subsequently obtained a US patent. From basalt, both discrete basalt fibers (BF) and continuous BF can be obtained [8]. The chemical structure of BF is similar to that of glass fibers, and it has excellent mechanical strength, thermal stability and chemical resistance [9,10]. When BF is filled into the material, it is necessary to consider the combination of BF and the matrix. When a weak interfacial interaction is formed between BF and the matrix, it may lead to the failure of the composite structure, thus limiting its service life and application range. Therefore, BF needs to be modified to improve the interaction between fibers and matrix [11,12,13,14,15].

Studies have found that mussel adhesive proteins can form strong adhesion to various substrates under certain conditions, and the key component is dopamine (DA). Inspired by this, many researchers began to use DA as a surface modifier for other materials [16,17,18]. The results show that polydopamine (PDA) can attach to almost all types of inorganic and organic surfaces. The self-polymerization of DA has the advantages of having a simple composition, mild reaction conditions and being suitable for further surface functionalization of various materials. The most attractive feature of DA modification is that the PDA coating can introduce active functional groups (hydroxyl groups, amino groups, etc.) without damaging the structure of the matrix. Its excellent adhesion provides an excellent opportunity for the functionalization of fillers. The excellent performance of PDA makes it a new kind of green modifier [19,20,21].

To date, there is a large body of work related to magnetorheological elastomers. In 1996, Jolly et al. [22] took the lead in conducting a more comprehensive study on magnetorheological materials, and a quasi-static, one-dimensional model is developed that examines the mechanical and magnetic properties of magnetorheological materials. Davis [23] has fully studied the modulus of the aligned MREs through calculation and predicted the optimal volume concentration of iron particles. Nowadays, the research of MREs has become an emerging research topic and has attracted more and more attention at home and abroad. According to the distribution of magnetic particles in the matrix, MREs can be divided into two categories, namely isotropic MREs and anisotropic (magnetic particle-aligned) MREs [24,25]. Stepanov et al. [26] investigated the viscoelastic behavior of isotropic MREs in an external homogeneous magnetic field and found a pseudo-plastic effect and a hundred-fold increase in the shear loss modulus of the MREs at small deformations. Qiao et al. [27] conducted experimental and simulation studies on the dynamic shear storage modulus and loss modulus of isotropic MREs, and proposed a new magneto-induced shear modulus model. Khimi et al. [28] compared the anisotropic MREs solidified under different magnetic fields, and pointed out that the magnetic particle chain becomes longer as the magnetic field strength increases during the solidification process. In addition, the size of magnetic particles also has a great influence on the magnetic response performance of MREs [29]. Of course, there are many reports on the optimal magnetic particle concentration of MREs [30,31]. There are also many studies on non-magnetic fillers of magnetorheological elastomers, mainly including reinforcing agents [32,33,34], plasticizers [35,36] and cross-linking agents [37,38]. However, there has been little research on other fillers. Li et al. [39] and Kumar et al. [40] fabricated MRE by adding multi-walled CNTs. The addition of a small number of CNTs can effectively increase the properties of MREs.

This paper focuses on the change in the mechanical properties of magnetorheological elastomers through the addition of modified fibers. It mainly includes tensile strength, tear strength, elongation at break, etc. Part of the performance of the sample under the magnetic field is also reflected in this paper. In this experiment, PDA was used to modify SiCB and BF, which effectively improved the interface bonding between SiCB and BF and the rubber matrix, and further improved the magnetorheological (MR) effect of MREs. The potential of BF and SiCB modified by PDA as green fillers was demonstrated by studying the mechanical properties and MR effects of the prepared MREs.

## 2. Experimental Methods and Testing

### 2.1. Materials

Natural rubber (NR) and butadiene rubber (BR) were purchased from Hainan Rubber Products Co., Ltd., Haikou, China. Carbonyl iron powder (average particle size 2.442 um) was purchased from Jiangsu Tianyi Superfine Metal Powder Co., Ltd., Huaian, China. SiCB was purchased from Jilin Kaiyu Biomass Development and Utilization Co., Ltd. (Changchun, China). Basalt fiber was purchased from Zhejiang Haining Anjie Composite Materials Co., Ltd., Jiaxing, China. Analytical grade reagents such as ZnO, Stearic acid, Tris (hydroxymethyl) aminoethane hydrochloride, dopamine hydrochloride and acetone were purchased from Sigma-Aldrich. Aromatic oil, Paraffin oil, Paraffin, sulfur, antioxidant 4010NA, antioxidant 4020, accelerator TMTD, accelerator CZ and accelerator DM were commercially available.

### 2.2. PDA Modified BF and BF^H^

**Figure 1:**
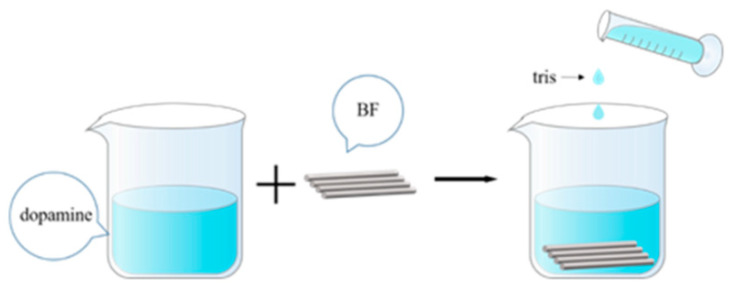
Flow chart of PDA modified BF.

PDA modified basalt fiber: Dissolved dopamine hydrochloride in a beaker with deionized water to prepare a solution with a concentration of 2 g/L. Dissolved Tris (hydroxymethyl) aminoethane hydrochloride in deionized water to prepare a 1 mol/L solution (Tris buffer solution). Basalt fibers pretreated with acetone and muffle furnace were added to dopamine solution followed by tris buffer at room temperature. The pH of the solution was adjusted to 8.5, and after 24 h of reaction at room temperature, it was taken out. Then it was rinsed with deionized water until it become neutral. Then, it was dried in an oven at 60 °C for standby and recorded as PDA-BF. The preparation process of PDA-modified BF is shown in Figure 1.

**Figure 2:**
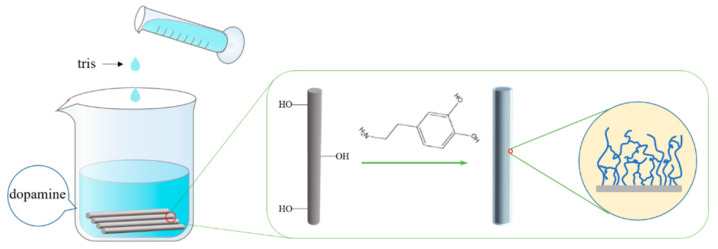
Mechanism diagram of PDA modified BF.

As shown in Figure 2, the mechanism analysis of PDA modified BF: dopaminequinone was synthesized in the presence of dissolved oxygen, alkaline buffer and an adequate supply of initial dopamine monomers. Then, dopaminequinone after oxidation, cyclization and rearrangement leads to 5,6-dihydroxyindole (A metastable intermediate). It then underwent deprotonation and intramolecular addition to generate amorphous PDA. The hydroxyl groups of PDA reacted with the exposed hydroxyl groups on the surface of BF to dehydrate, and PDA was grafted on the surface of BF to form a polydopamine coating.

**Figure 3:**
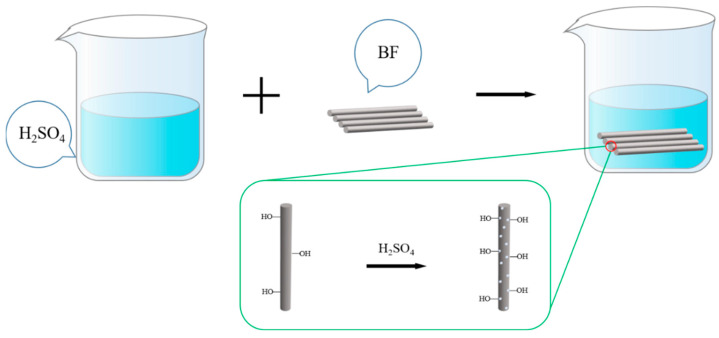
Schematic diagram of H_2_SO_4_ etching process of basalt fiber.

Preparation of BF^H^: in a fume hood, we prepared a 2 g/L sulfuric acid solution with 98% concentrated sulfuric acid. The pretreated and dried basalt fibers were added to this mixture. Then, they were ultrasonically oscillated in an ultrasonic machine for 10 min to make the basalt fibers evenly dispersed, reacted for 2 h at room temperature and then taken out. Rinsed in deionized water until neutral, and dried in an oven at 60 °C for later use. The process of H_2_SO_4_ etching basalt fiber is shown in Figure 3. Mechanism explanation of H_2_SO_4_ etching BF: BF contains a lot of SiO_2_ and some metal oxides (Fe_2_O_3_, Al_2_O_3_, MgO, CaO, Na_2_O and K_2_O, etc.). The SiO_2_ in BF does not react with H_2_SO_4_ and exists stably in BF, while the metal oxide in BF easily reacts with H_2_SO_4_ to generate sulfate which dissolves in the H_2_SO_4_ solution. Using this principle, H_2_SO_4_ was used to dissolve the metal oxides in BF, so that some protrusions and depressions were produced on the surface of BF, and the roughness and specific surface area of BF were increased. The operation of PDA-modified BF^H^ is the same as that of PDA-modified BF, denoted as PDA-BF^H^.

### 2.3. SiCB + PDA Synergistically Modified BF and BF^H^

**Figure 4:**
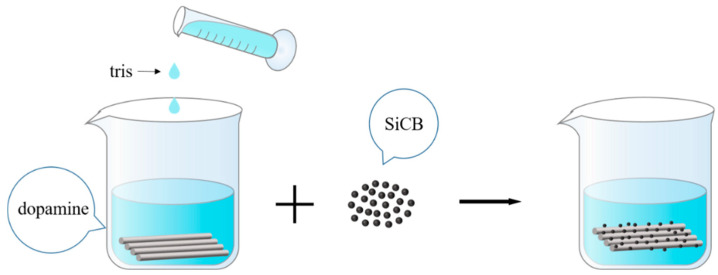
Flow chart of SiCB + PDA synergistic modification of BF.

(a) “Grafting” of SiCB to BF via PDA: The pretreated basalt fiber was added to the DA solution, then the tris solution was added to adjust the pH to 8.5, and SiCB was added after the reaction for 30 min. The mass of SiCB added was 20% of the mass of basaltic fiber, and it ultrasonically oscillated in an ultrasonic machine for 30 min. After a total of 24 h of reaction at room temperature, we poured out the solution, added deionized water, and after standing for 2 h, poured out the deionized water. The above operations were repeated until the solution was colorless and neutral, and then we dried the modified basalt fiber in a 60 °C oven for standby; denoted as PDA-BF-SiCB. The operation flow of SiCB + PDA synergistic modification of BF is shown in Figure 4.
(b) “Grafting” of SiCB to BF^H^ via PDA: The operation of SiCB + PDA modified BF^H^ is the same as that of SiCB + PDA modified BF, denoted as PDA-BF^H^-SiCB.

**Figure 5:**
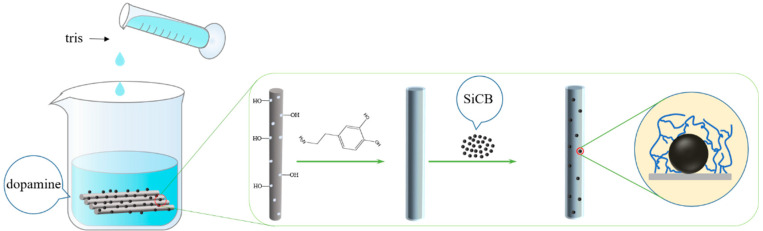
Mechanism diagram of SiCB + PDA synergistic modification of BF^H^.

As shown in Figure 5, the mechanism of SiCB + PDA synergistic modification of BF^H^: many tiny protrusions and depressions appeared on the surface of BF etched by H_2_SO_4_, which was beneficial to the infiltration of PDA. At the same time, more hydroxyl groups were introduced on the surface of BF^H^, which was beneficial to the graft modification of PDA. DA was oxidized and polymerized to PDA at room temperature, and PDA (acting as an intermediate bridge) was dehydrated by reacting with hydroxyl groups on the surface of BF^H^ and SiCB, respectively, to “graft” SiCB onto the BF surface.

### 2.4. Preparation of Magnetorheological Elastomer Precursors

According to the formula listed in Table 1, TY-7005 laboratory internal mixer (Jiangsu Tianyuan Test Equipment Co., Ltd., Yangzhou, China) was used to prepare MREs. The temperature of the mixer was set to 120 °C and the preheating was sufficient. The speed was set to 45 r/min, and the pressure of the top cover was set to 0.6 MPa. Added the cut block NR into the mixing chamber of the mixer for 1.5 min of plasticizing, then added BR and mix for 1.5 min. Added Carbonyl iron and basalt fibers (BF, BF^H^, PDA-BF^H^, PDA-BF-SiCB, PDA-BF^H^-SiCB) and mixed for 1 min; added paraffin and aromatic oil and mixed for 1.5 min; add stearic acid and ZnO and mix for 1.5 min. Then, adjusted the temperature of the internal mixer to 100 °C. Still used the rotating speed of 45 r/min and the pressure of the upper cover of 0.6 MPa. Added accelerator and antioxidant for mixing for 1 min; added sulfur mixing for 4 min. Finally, the composite was compressed for 8 min at 150 °C and 20 MPa to obtain the final MREs. Recorded as MREs/BF, MREs/BF^H^, MREs/PDA-BF, MREs/PDA-BF^H^, MREs/PDA-BF-SiCB, MREs/PDA-BF^H^-SiCB, respectively.

### 2.5. Microstructure Observation and Mechanical Properties Testing

A field emission scanning electron microscope (model: JSM-7900F, Japan Electronics Co., Ltd., Tokyo, Japan) was used to observe the microstructure of the BF under different treatment conditions and the tensile fracture surface of seven groups of MREs samples. Each sample was sprayed with a thin layer of gold and placed into the SEM. The microstructure of the samples was observed at an accelerating voltage of 5 kV. According to ISO 37:2005 standard and ISO 34-1:2004 standard, the mechanical properties of each group of samples were tested using an electronic universal testing machine (Model: AGS-X-100 kN, Shimadzu Instrument Co., Ltd., Tokyo, Japan). Data for each sample were the average of 5 tests. Tensile properties, Stress at 300% Strain, elongation at break and tear strength were tested, respectively. Additionally, a shore hardness tester (LX-A) was used to test its hardness.

### 2.6. Measurement of MREs’ Viscoelastic Property

Viscoelastic property of each set of samples was performed using an advanced rotational rheometer (Anton Paar, model: MCR 301). In the experiments, a disk specimen with a sample diameter of 20 mm and a thickness of 2 mm was used. The effect of different magnetic fields on the storage modulus of the samples was evaluated in shear mode. The range of the external magnetic field was 0–1000 mT. The driving frequency was fixed at 5 Hz and the dynamic strain amplitude was set as 0.03%. The experiments were carried out at room temperature.

## 3. Results and Discussion

### 3.1. Microstructure of Modified BF and MREs

**Figure 6:**
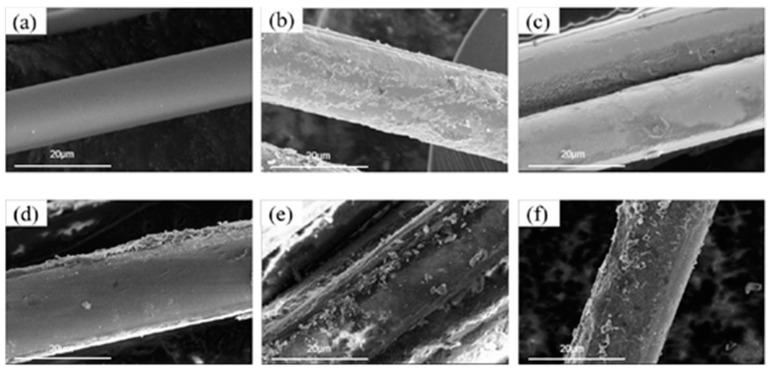
SEM images of BF under different treatment conditions at 20 μm: (**a**) BF, (**b**) BF^H^, (**c**) PDA-BF, (**d**) PDA-BF^H^, (**e**) PDA-BF-SiCB, (**f**) PDA-BF^H^-SiCB.

Figure 6 shows the SEM images of BF under different treatment conditions at 20 μm. From Figure 6a, it can be seen that the surface of the original BF was smooth, and no substance was attached. In Figure 6b, it can be seen that concave and convex defects appeared on the surface of BF^H^ after sulfuric acid etching, and the roughness and specific surface area increased. In Figure 6c, it can be seen that the PDA coating was grafted on the surface of BF. The surface was no longer smooth, and the roughness was increased compared with the original BF. From Figure 6d, it can be seen that PDA was well grafted on the surface of BF^H^, and its roughness was higher than that of PDA-BF. This is because the fiber is more prone to graft reaction with PDA after sulfuric acid etching, so PDA-BF^H^ presents a more irregular rough surface. From Figure 6e, it can be clearly seen that the bulk or granular SiCB was successfully “grafted” on the BF surface by PDA, and the surface roughness of PDA-BF-SiCB was greatly improved due to the addition of SiCB. Figure 6f shows that the surface of PDA-BF^H^-SiCB was successfully “grafted” onto SiCB by PDA, and its surface was the roughest. This is due to the joint action of H_2_SO_4_, PDA and SiCB, which effectively improved the fiber surface, and greatly improved the roughness and specific surface area.

**Figure 7:**
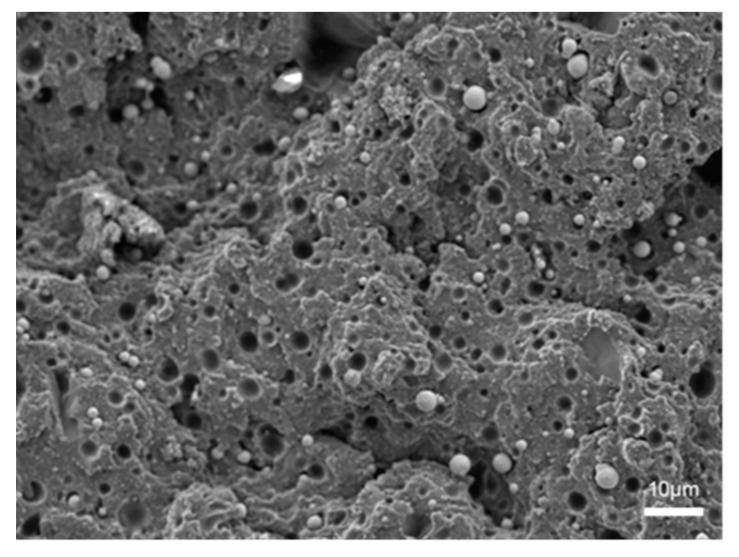
SEM image of tensile fracture surface of MREs.

**Figure 8:**
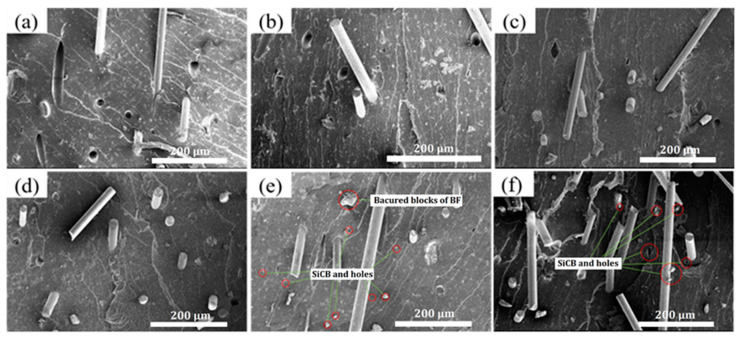
SEM images of rubber tensile fracture surface (**a**) MREs/BF (**b**) MREs/BF^H^ (**c**) MREs/PDA-BF (**d**) MREs/PDA-BF^H^ (**e**) MREs/PDA-BF-SiCB (**f**) MREs/PDA-BF^H^-SiCB.

Figure 7 is the SEM image of the tensile fracture surface of MREs at 10 μm. It can be seen that the carbonyl iron powder was dispersed on the surface of the matrix, and there was no other filler except iron powder. Figure 8 is the SEM images of the tensile fracture surfaces of the six rubber composites at 200 μm. It can be seen from the figure that BF in MREs/BF is easily pulled out due to the poor bond between the BF and the matrix. Compared with MREs/BF, the BF^H^ of MREs/BF^H^ has a stronger bond with the matrix because the convex and concave defects formed on the surface of BF after H_2_SO_4_ etching can play a certain “anchoring” role on the matrix and strengthen the interface strength. The fiber-matrix bonding in MREs/PDA-BF was further improved, and the interface between them became more compact because the PDA coating grafted on the fiber surface exhibits strong adhesion, which can form a stable and reliable interface between BF and matrix. There were obvious traces of H_2_SO_4_ etching and residual rubber on the surface of BF^H^ in MREs/PDA-BF^H^, and the interface between BF^H^ and the matrix was more compact and firmer, which is the result of the combined effect of PDA and H_2_SO_4_ etching. In MREs/PDA-BF-SiCB and MREs/PDA-BF^H^-SiCB, the bond between the fiber and the matrix was more compact and firmer. The SiCB that “pins” the matrix was scattered around the fiber, and there were basically no holes for fiber pulling out. It was the brittle fracture that was no longer the whole pull-out. This was the result of the synergistic effect of SiCB and PDA. The SiCB “grafted” on the fiber surface by PDA can pin the matrix well, and the SiCB that falls off the fiber (with a part of the PDA coating on the surface) can be used as an inorganic filler (adsorbs rubber molecular chains and forms a filler-rubber network) to strengthen the matrix; PDA provided a strong bond between the fiber and the matrix, forming a solid interface and increasing the interface strength between the two. It can be seen from the figure that there was a gap between the BF and the matrix in the MREs/BF, and the matrix was torn. In MREs/PDA-BF-SiCB, a large amount of rubber matrix remained at the root of BF. SiCB was scattered around BF, and BF was closely bound to the matrix, which is attributed to the joint effect of PDA and SiCB. The BF^H^ surface fiber of MREs/PDA-BF^H^-SiCB was rough, and there were residual SiCB and rubber matrix which were not dropped after tensile. The root of BF^H^ was closely bound to the matrix and the fracture section was rougher, which is the result of H_2_SO_4_ etching, PDA grafting modification and SiCB grafting through PDA.

### 3.2. Mechanical Properties of Composite Materials

**Figure 9:**
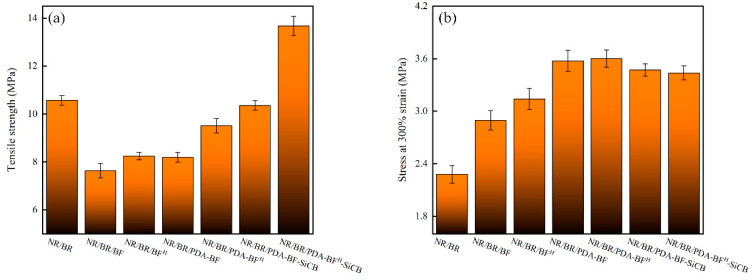
Tensile strength diagram (**a**) and Stress at 300% strain diagram (**b**) of each group of MREs.

Table 2 and Figure 9 show the tensile strength values, the stress at 300% strain values, tensile strength diagrams and stress at 300% strain diagrams of the seven MREs, respectively. Combined with Table 2 and Figure 9a, it can be seen that H_2_SO_4_ etching, PDA graft modification and SiCB + PDA synergistic modification all improved the tensile strength of MREs. The tensile strength of MREs/BF is the lowest because the surface of BF is smooth and easy to entangle and agglomerate; the poor bonding between BF and the matrix is difficult to effectively infiltrate, resulting in poor dispersion of BF in rubber; and the entangled BF is easy to form stress concentration points. The tensile strength of MREs/BF^H^ is higher than that of MREs/BF, because after BF is etched by H_2_SO_4_, good uneven defects appeared on the surface, which is a better “anchoring” effect on the rubber matrix, so it takes more energy to pull BF^H^ out. The tensile strength of MREs/PDA-BF and MREs/PDA-BF^H^ prepared from PDA grafted fibers is significantly higher than that of non-PDA treated rubber, because the PDA coating grafted on the fiber surface increases the active sites on the fiber surface. It is beneficial to the dispersion of fibers in the rubber, and the high adhesion of PDA strengthens the interfacial bonding between the fibers and the matrix, and enhances the interfacial strength between them. The tensile strength of MREs/PDA-BF-SiCB and MREs/PDA-BF^H^-SiCB prepared by PDA + SiCB synergistically modified fibers is further improved. The tensile strength of MRES/PDA-BF^H^-SiCB is the largest, reaching 9.58 MPa, which is 29.3% and 79.4% higher than that of MREs and MREs/BF, respectively. This is because the good “grafting” of SiCB on the BF^H^ via PDA action increases the specific surface area of BF, which increases its surface roughness, and SiCB can play a good role in “pinning” the matrix. This makes the fibers not easy to be pulled out, and also plays the role of sharing the stress on the material, so that the tensile strength of the MREs/PDA-BF^H^-SiCB is significantly improved. From Table 2 and Figure 9b, it can be found that both H_2_SO_4_ etching, and PDA graft modification have improved the stress at 300% strain of the composite materials. Among them, the stress at 300% strain of MREs/PDA-BF^H^ under the combined action of H_2_SO_4_ etching and PDA grafting was the largest, reaching 4.34 MPa. Because more active groups are introduced on the surface of BF^H^ and moderately convex and concave defects are etched after H_2_SO_4_ etched BF, which is beneficial to the infiltration and graft modification of PDA molecules. This enables the surface of BF^H^ to be grafted with a thick and uniform PDA coating, which strengthens the interaction between BF^H^ and rubber. Therefore, the interface between the two can be better combined, and the interface strength is enhanced, so that the MREs/PDA-BF^H^ exhibits better stress at 300% strain performance. The addition of SiCB slightly reduces the stress at 300% strain of MREs/PDA-BF-SiCB and MREs/PDA-BF^H^-SiCB, because the fiber surface is “grafted” with SiCB, which will slightly enhance the fiber dispersion, and the filler network formed between the fibers is weakened so that the stress at 300% strain of the composite material is slightly reduced. N. V. Shadrinov and S. I. Nartakhova tested the properties of carbon fiber and basalt fiber-filled nitrile rubber [41]. Some mechanical properties of composite materials are shown in Table 3. It can be seen that the tensile strength of the composite material without iron powder is higher because the addition of iron powder usually leads to the destruction of the rubber molecular network. However, the tensile strength of MREs/PDA-BFH-SiCB reaches 9.58 MPa, which has a great increase in mechanical properties compared with other magnetorheological elastomers [42]. Chen et al. [43] tested the tensile strength of natural rubber-based anisotropic magnetorheological elastomers with different carbon black content, and their mechanical properties are also shown in Table 3. In Table 4, sample 1 contains 33% volume fraction of magnetic particles without adding carbon black, and sample 2 contains 33% volume fraction of magnetic particles and 4% volume fraction of carbon black. Sample 3 contained a 33% volume fraction of magnetic particles and a 7% volume fraction of carbon black. The comparison can prove the great potential of BF modified by the method described in this paper as a filler. At the same time, the coupling effect of magnetic particles, nano-reinforced particles, and fiber reinforcement enhances the mechanical properties of the rubber matrix in this study.

**Figure 10:**
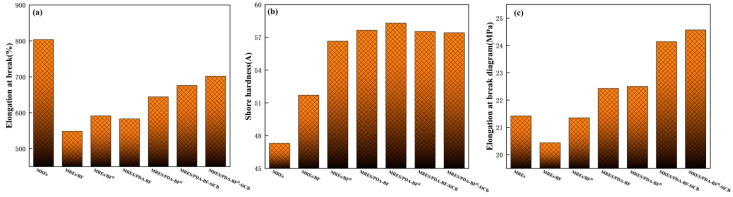
Elongation at break diagram (**a**) Shore hardness diagram (**b**) Tear strength diagram (**c**) of each group of MREs.

Table 4 and Figure 10 show the elongation at break values, hardness values and tear strength values and hardness graphs, elongation at break graphs and tear strength graphs for the 7 MREs, respectively. According to Table 4 and Figure 10a, it can be seen that the elongation at the break of the material added with BF is significantly smaller than that of the original rubber MREs. This is because BF is an inorganic material that exhibits strong rigidity, hindering the movement of rubber molecular chains and reducing the tensile length of the material. However, the three methods of H_2_SO_4_ etching, PDA graft modification and SiCB + PDA synergistic modification increased the elongation at the break of the material. The best elongation at break is 701.8% for MREs/PDA-BF^H^-SiCB, which is 28.0% higher than that of MREs/BF. Under the joint action of the three, the surface roughness and specific surface area of BF^H^ increase and the active sites also increase. This makes the combination of BF^H^ and the matrix more stable, and the interface strength between the two is greatly improved. The BF^H^ can better disperse the load on the matrix, and these changes will hinder the pull-out of the BF^H^. According to Table 4 and Figure 10b, the addition of BF improves the hardness of MREs/BF compared with MREs, because the addition of BF as an inorganic filler can increase the hardness of the material. Both the etching of BF by H_2_SO_4_ and the graft modification of BF by PDA have improved the hardness of the material. It is because the etching of H_2_SO_4_ and the grafted PDA coating play a good role in reducing the agglomeration and entanglement of BF, strengthening the BF skeleton, and strengthening the interface strength between BF and rubber, thereby increasing the hardness of the material. Additionally, under the combined effect of the two, the hardness of MREs/PDA-BF^H^ reaches the maximum, which is 58.3 A, which is 23.3% and 12.8% higher than that of MREs and MREs/BF, respectively. However, the effect of SiCB on the hardness of the composites is not great. The hardness of MREs/PDA-BF^H^-SiCB and MREs/PDA-BF-SiCB is slightly lower than that of MREs/PDA-BF^H^ and MREs/PDA-BF, respectively. This is because the addition of SiCB will promote the dispersion of fibers to a certain extent and weaken the filler network formed between the fiber skeletons, thus slightly reducing the hardness of the material. According to Table 5 and Figure 10c, it can be seen that the three methods of H_2_SO_4_ etching, PDA graft modification and SiCB + PDA synergistic modification can enhance the tear strength of the material. Among them, MREs/PDA-BF^H^-SiCB has the highest tear strength of 24.07 kN/m, which is 15.1% and 20.7% higher than MREs and MREs/BF, respectively. This is the result of the combined action of H_2_SO_4_, PDA and SiCB. First, the surface roughness of BF after H_2_SO_4_ etching increases, and the active sites increase, which is beneficial to the grafting of PDA. Secondly, the PDA coating grafted on the surface of BF^H^ is conducive to the dispersion of BF^H^, and its excellent adhesion is beneficial to enhance the interface strength between BF^H^ and the matrix and the “grafting” of SiCB to BF^H^; Furthermore, the SiCB on the surface of the BF^H^ greatly increases the comparative area and surface roughness of the BF^H^, and the SiCB has a strong “pinning” effect on the matrix, hindering the propagation of cracks.

### 3.3. MR Effect

**Figure 11:**
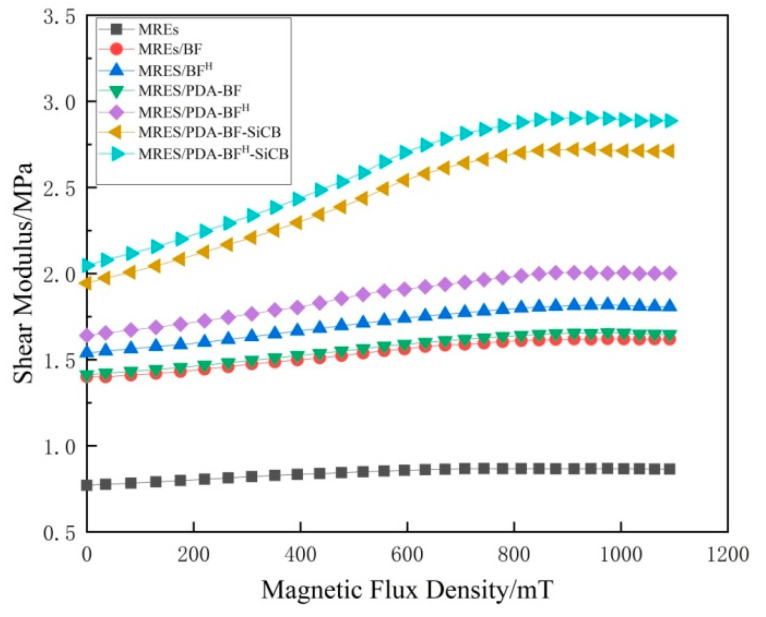
Shear modulus of samples of each group of MREs under different magnetic flux density.

The Shear modulus of seven groups of samples under various magnetic fields from 0 to 1000 mT was measured. As shown in Figure 11, it can be seen from the figure that the shear modulus of the MREs increases with the increase in the magnetic field strength before reaching the saturation magnetic field. This is because the dipole interaction of magnetically polarized particles is expanded by the influence of the magnetic field. Therefore, the shear modulus changes with the applied magnetic field. Table 5 lists the performance of seven groups of samples under the magnetic field. In this table, $G_{0}$ notes the MREs samples’ zero field modulus, $\Delta G_{\max}$ denotes the saturated field-induced modulus, $\Delta G_{\max}/G_{0}(\%)$ denotes the relative MR effect. It can be seen from the figure that the Shear modulus increase significantly after adding BF because the presence of BF will increase the damping of the motion of the rubber molecular chain. The BF etched by H_2_SO_4_ has a rougher surface, which also makes the damping greater. Therefore, the storage modulus of the sample filled with BF^H^ is higher than that of the MREs sample filled with BF. Comparing MREs/PDA-BF with MREs/BF and MREs/PDA-BF^H^ with MREs/BF^H^, it can be seen that the PDA modification has little effect on the storage modulus of the material because this modification does not create additional damping and interaction. It is worth noting that comparing the samples MREs/PDA-BF^H^-SiCB and MREs/PDA-BF^H^, we can find that the MREs with SiCB added, although the volume ratio of magnetic particles is the same, the change of the storage modulus is significantly higher than that without the addition of SiCB. The MR effect of MREs/PDA-BF^H^ is 22.26%, while that of MREs/PDA-BF^H^-SiCB is 44.01%. In comparison, although the two groups of samples contain the same amount of fiber; the MR effect of MREs/PDA-BF^H^-SiCB is nearly doubled compared with that of the sample MREs/PDA-BF^H^. This shows that SiCB has greatly improved the MR effect of MREs with the addition of modified BF. Because the good “grafting” of SiCB on BF^H^ through PDA increases the specific surface area of BF, and it is well combined with the rubber molecular chain to form a new bond, which makes under the action of a magnetic field, the coupling interaction of magnetic particles have a greater impact on the internal molecular structure of the material. Therefore, the MR effect can be improved. The reason for the difference in the MR effect between MREs/PDA-BF-SiCB and MREs/PDA-BF is the same, because SiCB makes more bonding inside the composite material, which enlarges the influence of the magnetic field on the rubber matrix and improves the MR effect.

## 4. Conclusions

BF can be modified by H_2_SO_4_ etching, PDA grafting or PDA and SiCB co-modification to improve its performance as filler. The overall mechanical properties of the composite prepared by modified BF as filler are improved compared with MRES/BF. It is demonstrated that the PDA coating promotes the dispersion of fillers and enhances the surface activity of SiCB. It effectively solves the problem that SiCB is difficult to be effectively penetrated by rubber due to its strong polarity and enhances the interfacial force between the filler and the matrix. Among them, MREs/PDA-BF^H^-SiCB has the best overall performance. Its tensile strength, tear strength, and Stress at 300% strain reach 9.58 MPa, 24.07 kN/m, and 4.13 MPa respectively, which is 29.3%, 15.1% and 50.7% higher than ordinary MREs. In addition, after adding PDA and SiCB synergistically modified BF^H^ to MREs, the MR effect will be greatly improved due to the increase in their internal bonding. The MR effect of the new MREs (MREs/PDA-BF^H^-SiCB) prepared in this way can be more than three times that of traditional MREs.

## Figures and Tables

**Table 1:** MREs formulation.

Ingredients	Amounts (phr)
NR/BR	70/30
Carbonyl iron	200
Filler	X
ZnO	6
Stearic acid	1
Paraffin oil	6
Antioxidant 4010NA	2
Antioxidant 4020	2
Accelerator TMTD	0.2
Accelerator CZ	1.5
Accelerator DM	0.4
Sulfur	1.2

X represents the change in the number of fillers.

**Table 2:** Tensile strength values and Stress at 300% strain values of each group of MREs.

Materials	MREs	MREs/ BF	MREs/ BF^H^	MREs/ PDA-BF	MREs/ PDA-BF^H^	MREs/ PDA-BF-SiCB	MREs/ PDA-BF^H^-SiCB
Tensile Strength (MPa)	7.41	5.34	5.57	5.74	6.66	7.25	9.58
Stress at 300% Strain (MPa)	2.74	3.49	3.77	4.30	4.34	4.16	4.13

**Table 3:** Properties of elastomeric fiber composites.

Materials	Tensile Strength (MPa)	Elongation at Break (%)	References
BNKS-18	14.8	525.9	[41]
BNKS-18 + 2.5 BF	13.2	470.1	
BNKS-18 + 5.0 BF	14.5	397.8	
BNKS-18 + 7.5 BF	14.3	416.9	
BNKS-18 + 10.0 BF	14.4	416.7	
MRE (without carbon black)	2.37	-	[43]
MRE (4% carbon black)	3.25	-	
MRE (7% carbon black)	3.52	-	

**Table 4:** Elongation at break values, Shore hardness values and Tear strength values of each group of MREs.

Materials	MREs	MREs/ BF	MREs/ BF^H^	MREs/ PDA-BF	MREs/ PDA-BF^H^	MREs/ PDA-BF-SiCB	MREs/ PDA-BF^H^-SiCB
Elongation at Break (%)	803.48	548.33	591.23	582.85	644.29	676.04	701.84
Shore Hardness (A)	47.3	51.7	56.7	57.6	58.3	57.5	57.4
Tear Strength (kN/m)	20.92	19.94	20.85	21.93	22.00	23.64	24.07

**Table 5:** The MR effect of each sample.

Sample	$G_{0}$ (MPa)	$\Delta G_{\max}$ (MPa)	$\Delta G_{\max}/G_{0}$ (%)
MREs	0.77	0.098	12.73
MREs/BF	1.401	0.22	15.7
MREs/BF^H^	1.539	0.279	18.12
MREs/PDA-BF	1.412	0.244	17.28
MREs/PDA-BF^H^	1.64	0.365	22.26
MREs/PDA-BF-SiCB	1.944	0.779	40.07
MREs/PDA-BF^H^-SiCB	2.047	0.856	41.82

## Data Availability

The datasets used or analyzed in the current study are available fromthe corresponding author on reasonable request.
